# Notes and News

**Published:** 1898-07-02

**Authors:** 


### Notes and News.

(Collected and Communicated.)

The warning note common at this season of the year against poisonous mushrooms is being sounded in the daily press.

On the 18th ult. the Lord Mayor and Lady Mayoress laid the foundation-stone of the new chapel belonging to the City of London Asylum at Stone.

The Tangier Sanitary Council expresses a fear lest the new lazaretto for Mecca pilgrims on Mogador Island may prove a source of infection, since other buildings and the Government convict prisons exist on the island.

The authorities of the Prussian Baltic bathing places are denying the rumours which have been current that leprosy exists in the seaside resorts on the Baltic coast. The reports arose from the fact of an official report being issued relative to the lepers' colony in the Prussian district of Memel.

In accordance with the desire expressed by some of the contributors to the Huxley Memorial Fund, replicas of the design of the Memorial Medal may now be purchased by subscribers to the fund. Prices and particulars may be obtained from the Hon. Secretary to the Huxley Memorial Committee, at the Royal College of Science, South Kensington, where these replicas will be on view until the end of September.

Mr. E. G. Annis, Medical Officer of Health for Huddersfield, referring in his report to certain cases of puerperal fever, says that he has asked all the medical men in that town to supply him with the numbers of the confinements attended by them during the last year or two, and that as a result of his inquiries he finds that roughly one half of the accouchements in Huddersfield are attended by midwives, and the other half by medical men. The homes of some of the midwives showed such "evidences of neglect, dirt, filth, and a general squalid condition" that it seemed to him a wonder that puerperal fever was not even more common than was the case, considering the number of cases attended by them.

On Saturday afternoon, July 9th, the annual meeting of the United Hospitals Athletic Sports will be held at Stamford Bridge. The shield is at present in the possession of St. Mary's.

At the successful matinée given at the Vaudeville Theatre, in aid of the Charing Cross Hospital Convalescent Home at Limpsfield, Surrey, on the 9th ult., a very popular part of the programme consisted in the appearance of the Gordon Highlanders.

It is a curious and interesting spectacle to see Sir Walter Foster, a leading representative of medicine in the House of Commons, fighting day after day in committee against the compulsory clauses of the Vaccination Bill.

The Prince of Wales has appointed Colonel C. W. B. Bowdler, at present Deputy Commissioner No. 1 District, to succeed Colonel Sir Edward Talbot Thackeray, V.C., as Commissioner of the St. John Ambulance Brigade.

H.R.H. Princess Christian opened on the 21st ult. the new ward which has been added to the Windsor and Eton Royal Infirmary, to commemorate the Diamond Jubilee, the funds for this having been raised locally.

The new gown to be worn by Fellows of the Royal College of Surgeons on ceremonial occasions is of black stuff with crimson satin facings of a regulation shape and shade. Fellows who are also graduates of any University may use the gowns representing their degrees on the occasions when the Fellow's gown would otherwise be worn.

The June number of the *Nineteenth Century* savours much of medicine. Lady Meath contributes a delightful article on "A Woman's Hospital in Morocco," Dr. Marion Hunter gives her experiences whilst "Fighting the Plague at Poona," and Mrs. King writes about "Death and Torture under Chloroform." Sir Henry Thompson crowns this medical number by an article entitled, "Why Vegetarian?" a reply to critics.

The General Medical Council has failed in its action against John Hamliton for falsely pretending to and using the title of doctor of medicine, he being an unregistered practitioner. It was proved that a person visiting him paid him two guineas for medicine, and that there was a brass plate on the door with the words "John Hamilton, M.D. New York." It must be remembered, however, that in this case the words "New York" were appended to the letters "M.D." This is an important matter. The Medical Act imposes a penalty on any person wilfully and falsely using the title (among others) of "Doctor of Medicine," but it goes on to add "or other title, addition, or description implying that he is a registered person, or that he is recognised by law as . . . . a practitioner in medicine." Now it is clearly arguable that as the M.D. of New York is not a registerable degree, and that as its possessor is not recognised by law as a practitioner in medicine, there is no infringement of the Act in its assumption. It is quite different, however, with the use of the title M.D. pure and simple, and that is the question which this case does nothing to decide, namely, Are the letters M.D. a title, addition, or description "implying" that the possessor is "recognised by law as a practitioner in medicine"?

THE treasurer of St. Mark's Hospital for Fistula, City-road, has received a cheque for £1,000 from Mrs. Henty for the endowment of a bed to be named the "Julia Henty Bed."

H.R.H. THE PRINCESS OF WALES has consented to receive purses at the opening of the Pfeiffer Wing of the London School of Medicine for Women on July 11th, and parents wishing their children to participate in the purse presentation are requested to communicate with the secretary of the School at 30, Handel Street, Brunswick Square, W.C.

AT a meeting of the "Richmond Local Memorial" to the late Duchess of Teck, it was announced that subscriptions amounted to £355, and the Mayor put forward two schemes for the memorial—(1) To endow a bed in the name of the Princess in the Royal Hospital, Richmond, at a cost of £750; and (2) to erect a drinking fountain just inside the park, with a medallion of the Princess. The latter scheme was unanimously adopted.

WE regret to find that when the bye-laws for the regulation of street noises, which had been proposed by a committee of the London County Council, came before the full Council for approval they were referred back, on the pretence that they would interfere with the activities of the gentle costermonger. The sick, whether rich or poor—and the poor are far more numerous and far less protected than the rich—are to be sacrificed in order that those costermongers and newsvendors who happen to be blessed with loud voices may take the bread out of the mouths of their feebler or more law-abiding fellows.

THE Petroleum Committee has decided by eight votes to six to recommend the raising of the flash-point from 73 deg. to 100 deg.

THE warrant constituting the Royal Army Medical Corps has been approved by Her Majesty, and will be published in the Army orders for July.

IN connection with the East London Hospital for Children, Shadwell, Viscountess Falkland has undertaken to form a ladies' association. The trustees of Smith's Charity have sent a donation of £100 to the hospital.

A "REPROACH and an appeal" has been directed to the *Daily Graphic* by a correspondent who calls attention to the neglect on the part of the public to send to the military hospitals any of those presents of fruit and flowers which at this season are offered in such profusion to the civil hospitals. It is stated on unimpeachable authority that, year in, year out, not "a single flower or a solitary strawberry reaches one of our largest military hospitals." The small public interest taken in the sick soldier is one which is thoroughly recognised in hospital circles, and it is right that attention should be called to such apathy. A circle of ladies known as the "Jackanapes" Society sends presents of warm bed-jackets and furnishes underclothing for the use of soldiers leaving hospital, and their kindness in providing such helps to the sick soldier is much appreciated. The society was formed by Mrs. Eden, sister of Mrs. Ewing, who has written such charming stories of soldier-life. Beyond the assistance of this helpful little society, the sick soldier meets with practically no assistance from sources outside the hospitals.

